# Identifying barriers and facilitators to ambulance service assessment and treatment of acute asthma: a focus group study

**DOI:** 10.1186/1471-227X-14-18

**Published:** 2014-08-03

**Authors:** Deborah Shaw, Aloysius Niroshan Siriwardena

**Affiliations:** 1 Clinical Audit and Research Department, East Midlands Ambulance Service NHS Trust, East Division Headquarters, Cross O’Cliff Court, Lincoln LN4 2HL, England; 2 Community and Health Research Unit, School of Health and Social Care, College Social Science, University of Lincoln, Brayford Pool, Lincoln LN6 7TS, England

**Keywords:** Asthma, Prehospital, Emergency medical services, Paramedic, Clinical guidelines

## Abstract

**Background:**

Acute asthma is a common reason for patients to seek care from ambulance services. Although better care of acute asthma can prevent avoidable morbidity and deaths, there has been little research into ambulance clinicians’ adherence to national guidelines for asthma assessment and management and how this might be improved. Our research aim was to explore paramedics’ attitudes, perceptions and beliefs about prehospital management of asthma, to identify barriers and facilitators to guideline adherence.

**Methods:**

We conducted three focus group interviews of paramedics in a regional UK ambulance trust. We used framework analysis supported by NVivo 8 to code and analyse the data.

**Results:**

Seventeen participants, including paramedics, advanced paramedics or paramedic operational managers at three geographical sites, contributed to the interviews. Analysis led to five themes: (1) guidelines should be made more relevant to ambulance service care; (2) there were barriers to assessment; (3) the approach needed to address conflicts between clinicians’ and patients’ expectations; (4) the complexity of ambulance service processes and equipment needed to be taken into account; (5) and finally there were opportunities for improved prehospital education, information, communication, support and care pathways for asthma.

**Conclusions:**

This qualitative study provides insight into paramedics’ perceptions of the assessment and management of asthma, including why paramedics may not always follow guidelines for assessment or management of asthma. These findings provide opportunities to strengthen clinical support, patient communication, information transfer between professionals and pathways for prehospital care of patients with asthma.

## Background

In the United Kingdom (UK) there are over 5.4 million people with asthma. In 2008/9 there were almost 80,000 emergency hospital admissions and over 1000 deaths from asthma [1,2]. Between 26% and 59% of patients with acute asthma requiring hospitalisation are attended by ambulance services [3-5].

Ambulance clinicians, including paramedics (staff registered with the Health Professions Council, the UK professional regulatory body), advanced paramedics with additional specialised training in primary care or trauma (emergency care practitioners, community paramedics and critical care paramedics), and emergency care technicians or assistants (unregistered healthcare staff with basic emergency and ambulance driving training) provide important assessment and potentially life-saving treatment at the patient’s home and, if necessary, provide transport to hospital for further treatment according to national guidelines.

Ambulance services in the UK use national guidelines from the Joint Royal Colleges Ambulance Liaison Committee (JRCALC). At the time of the study JRCALC UK Ambulance Service Clinical Practice Guidelines 2006, covering general, condition specific, and drug guidelines for asthma were in use (Table 1) [6]. The JRCALC and related British Thoracic Society [6,7] guidelines emphasise the importance of early initial assessment of severity of asthma followed by specific treatments which can improve symptoms, enhance outcomes and prevent avoidable deaths from asthma [8].

**Table 1:** **JRCALC guidelines for asthma 2006**[6]

**Assessment**	**Management**
Assess ABCD (airway, breathing, circulation, disability) for severity of asthma.	- Correct ABCD.
Check PEFR if practicable.	- Administer high dose oxygen.
Monitor ECG and pulse oximetry.	- Commence transfer to definitive care.
Reassess to measure improvement in peak flow or chest air entry.	- Administer salbutamol via oxygen driven nebuliser at 6-8l/minute.
	- In acute or severe life threatening cases add ipratropium bromide via nebuliser.
	- Obtain intravenous access if possible.
	- If no clinical improvement after 5–10 minutes repeat salbutamol nebuliser, consider continuous nebulised salbutamol and add ipratropium bromide nebuliser if not given previously.
	- Administer hydrocortisone intravenously.
	In life threatening asthma:
	Administer adrenaline intramuscularly.

PEFR = Peak Expiratory Flow Rate; ECG = Electrocardiograph.

Guideline recommendations include performing peak expiratory flow rate (PEFR) and pulse oximetry measurements which provide important objective assessments of asthma severity[9,10] and help to determine treatments used and need for hospitalisation[11,12]. Nebulised or inhaled beta-agonists (most commonly salbutamol) and oxygen are the main treatments advocated [6,7].

Although prehospital assessment and treatment for asthma provided by ambulance clinicians can improve outcomes, significant shortfalls in adherence to recommendations have been demonstrated in published audits of ambulance services: although these have shown higher rates of treatment with nebulised beta-agonists for people with asthma (46-93%), there are lower levels of PEFR (11-49%) and pulse oximetry measurement (80-89%) performed [4,5,13,14]. Ongoing national audits of prehospital asthma care involve sampling data from ambulance records on clinical performance indicators developed for asthma twice yearly, benchmarking services using funnel plots and developing interventions to improve care [14,15].

Studies into barriers to practitioner adherence to guidelines for patient care have pointed to clinician disagreement with guidelines, scepticism about whether they improve patient outcomes, and inertia or lack of motivation to change practice [16]. Studies aimed at changing behaviour emphasise that change requires comprehensive approaches including an understanding of those determinants of practice which are barriers and enablers to improvement[17] and the use of a range of reinforcing strategies to bring about desired change [18].

There has been little previous research investigating barriers to ambulance clinicians’ adherence to national guidelines for asthma and how these might be overcome. We aimed to explore ambulance clinicians’ attitudes, perceptions and beliefs about prehospital assessment and management of asthma and to identify factors which might prevent or enable adherence to guidelines.

## Methods

### Design

We were interested in participants’ attitudes, perceptions and beliefs formed through their experiences of assessment and treatment of patients with asthma so we used a qualitative design adopting an interactionist approach [19,20].

### Setting and sample

The study was conducted in a single regional ambulance service providing emergency and unscheduled care across five large counties in England. The service responds to over 616,200 emergency calls per year [21], approximately 4,500 of which relate to calls for patients with asthma (personal communication).

We used purposive sampling to recruit paramedics to the study. Although patients may be attended by registered paramedics or unregistered emergency care technicians or assistants, it is registered clinicians who lead care management for most patients with asthma who call an ambulance. To gather a variety of views we conducted focus groups in three different counties with staff at different levels of experience and seniority including paramedic team leaders (PTLs), operational managers (OMs) and community paramedics (CPs). Ambulance clinicians were recruited through advertisements posted in the organization’s in-house magazine, notice boards, intranet, through information leaflets and via local ambulance managers. The information included how interested clinicians were to contact the investigator to participate.

Approval for the study was granted by the NHS Research Ethics Service and Research Governance approval from the Trust in which the study took place. Informed, written consent was obtained from all participants.

### Data collection

We used focus groups (FG) to encourage interaction and discussion between participants, eliciting as wide a range of views and experiences as possible and enabling a deeper exploration of issues than would be possible through individual interviews or survey responses [22]. Data collection continued until no new themes emerged suggesting that data saturation had been achieved. Had new themes emerged during the third focus group more group interviews would have been conducted.

Each group interview lasted up to two hours. A topic guide used to generate discussion and modified as the study progressed (Additional file 1). FG1 was led by a clinician researcher (AS) while FG2 and FG3 were led by the non-clinical researcher (DS), both based in the Trust.

### Data analysis

Digital recordings of the focus groups were transcribed and anonymised by one of the researchers (DS) and an administrator. Analysis was supported by QSR NVivo 8 [23]. We used framework analysis to analyse the data using stages of familiarisation; identifying a thematic framework; indexing; charting; mapping and interpretation [24]. Transcripts were read, reread and coded line by line by one author (DS). An initial framework was constructed with three broad a priori themes based on the study objectives relating to guidelines, facilitators and barriers. New themes were added as data were coded and categorised (by DS/NS) through an iterative process of review and discussion [25]. No new themes emerged during the final focus group, suggesting that data saturation was achieved.

## Results

Three focus groups were conducted in three English counties including 17 participants (9, 5 and 3 participants) in total (Table 2).

**Table 2:** Participant characteristics

	**Focus group 1**	**Focus group 2**	**Focus group 3**
**Number**	9	5	3
**Sex**	2 female	3 female	1 female
	7 male	2 male	2 male
**Experience (years)**	5 to 35	9 to 14	2 to 16
	7 PTLs		
**Clinician type**	1 OSM	5 paramedics	2 CPs
	1 OSM/CP		1 Paramedic

PTL = paramedic team leader; OSM - operational senior manager, CP = community paramedic.

During initial analysis 60 codes were identified which we grouped into the following themes: (1) guidelines should be more relevant to ambulance service care; (2) there were barriers to assessment; (3) the approach needed to address conflicts between clinicians’ and patients’ expectations; (4) the complexity of ambulance service processes and equipment needed to be taken into account; and (5) there were opportunities for improved prehospital education, information, communication, support and care pathways for asthma. Themes and subthemes are shown in more detail below.

Themes and subthemes:

1. Guidelines should be more relevant to ambulance service care.

Guidelines need to be credible, clearer and simpler.

Guidelines should be applicable and practical in the prehospital environment.

2. Barriers to assessment.

Ambivalence to assessment.

Barriers to recording assessments.

Poor interprofessional communication at handover.

3. Conflict between clinicians’ and patients’ expectations.

Varying patient knowledge and skill.

Unmet patient expectations.

Patient barriers of language and diversity.

4. Complexity of ambulance service processes and equipment.

Difficulties diagnosing or confirming asthma.

Complex prehospital care processes.

Equipment burden.

5. Improved education, information, communication, support and pathways.

Practitioner education and patient information.

Interprofessional communication and clinical support.

Better care pathways.

### Guidelines should be more relevant to ambulance service care

#### *Guidelines need to be credible, clearer and simpler*

The credibility of guidelines was important to participants. Clinicians stated they did not always feel bound to carry out every process set out in JRCALC guidelines as they perceived them to be ‘guides’ to treatment rather than a rigid set of protocols. This, they felt, provided more scope to use their clinical judgement. Participants indicated they were more likely to adhere to guidelines that were clear, easy to follow and in a logical order, comprising elements they considered were beneficial to patients or necessary for the treatment pathway. JRCALC guidelines for other conditions were considered better than those for given for asthma, which were described as being ‘too wordy’, for use in the ambulance setting.

*When we moved to guidelines it gave you more scope to make your own judgement… some staff prefer to make a judgement, [because] not all patients quite fit in the right boxes…***[FG3/P1]**

*I think asthma is…* [a condition] *where we perhaps would do it in a different order. With the cardiac arrest algorithm you follow near enough to the word because obviously being best practice and research and everything and is the best way to carry out things. It’s easy and quick to follow rather than a load of text that you have to read through and then.’***[FG2/P3]**

### Guidelines should be applicable and practical in the prehospital environment

A key factor in a guideline’s credibility was its relevance to the prehospital environment and practicability. Some participants expressed that they felt the asthma guideline was more suited to hospital and primary care environments where equipment was readily to hand, there were more staff available to assist in treatment and transportation was not a consideration. They pointed out that the guidelines were less practicable in the emergency prehospital environment because ambulance staff worked alone or in pairs, were required to carry equipment from and to the vehicle, had to consider the safety of the environment they found themselves in, and distance to hospital. The practical difficulties of the ambulance environment therefore affected the order that guideline processes were carried out and whether all elements were delivered.

*I think treating patients there and treating patients in hospital are completely different. Sometimes you can’t stay in the environment. Sometimes you’re a few minutes from hospital… they’re so unwell you’ve put them on the ambulance, you’ve gone and you might be 2 minutes down the road, you haven’t done the whole thing because you’ve just not had the time, the environment wasn’t safe, they didn’t understand what you were trying to do, they didn’t want your help. There are so many other things that make it a little bit more difficult than it would be in a nice comfortable hospital cubicle where everything’s to hand. You know you might be, I don’t know, in the middle of site, shimmying down in a silo. There are loads of different environments that you end up stuck in that you just can’t do it. Either it’s not safe…you know you’re on your own…There are quite a few different areas that alter your judgment that would be different than if you were in your doctor’s surgery or hospital cubicle really.***[FG3/P1]**

I think everybody understands the nicety of getting a peak flow before [treatment].’

*‘And you consider it. You do consider it when you go in but the practicalities…[of performing a peak flow are problematic.’***[FG2/P3/P2]**

### Barriers to assessment

#### *Ambivalence to assessment*

Participants were ambivalent to assessment and often prioritised treatment over assessment. They considered some assessments to be less important than others in certain circumstances. For instance, they had been trained to correct airway and breathing problems immediately where a patient was ‘struggling to breathe’. Some felt that measuring pre-treatment PEFR or pulse oximetry, could delay treatment, was of little benefit, or might even be detrimental to a patient having difficulty breathing. These objective assessments were more likely to be performed before treatment if clinicians felt that the patient’s condition was stable or if the patient was more likely to be left at home. Many participants believed that a PEFR was not helpful if a patient’s previous best value was not available for comparison. These attitudes and beliefs meant that clinicians were likely to fall back on other less objective assessments such as the patient’s apparent breathing difficulty.

*I think another aspect from the clinician’s point of view, or how we train them, is that you can look at the patient to decide, “You’re ill” or “Yes, I can afford to do one or two tests before I can start making my mind up and then look at the measurements that comes off the machine.” The first part comes with experience.***[FG3/P2]**

#### *Barriers to recording assessments*

Some participants believed that acquisition of pre-treatment objective assessments was more for the purposes of clinical audit than patient care. It was suggested that the seemingly low recording rates of PEFR and pulse oximetry were in part due to under-recording. It was pointed out by participants that it was difficult to complete a clinical record (termed a patient report form or PRF) while transporting a patient being treated and monitored, and details of assessments could be missed when recording information after the event. The design of paper and electronic PRFs, with limited space on paper forms to record assessments and electronic recording systems being complex to use, was also seen as a barrier to completion.

*I think if you break it down, there’s us treating the patients and us recording what we’ve got. Everything we are trained for is we treat patients first that’s the be all and end all. Writing about it is a nice to have…‘If you’ve got a really poorly patient the last thing you’re doing is writing a PRF… So it’s the copying of information from the back of your glove or what you’ve got in your head, back on to the paper. That’s where I think some of the problems start.***[FG3/P2]**

I wonder if the [electronic records], if they have actually done what they should be doing, they’ve just not recorded it, because they can’t find it.

*I think people who’ve used the system, and they use it very well, but it still doesn’t necessarily lend itself very well to how we work.***[FG3/P2/P1]**

#### *Poor inter-professional communication at handover*

There was also a perception among some participants that hospital staff were not interested in the measurements obtained by ambulance crews and this was an added disincentive to carrying these out.

*…we usually do a pre-alert so you usually have a doctor standing by to take over straight away. But no they very rarely ask* [for the PEFR reading]*.***[FG2/P1]**

### Conflict between clinicians’ and patients’ expectations

#### *Varying patient knowledge and skill*

Participants believed that patients’ knowledge of self-care and treatment regimens varied, with some patients less aware of what their prescribed medications were for, or when to access services during deteriorations in their condition. Some patients with worsening asthma called for ambulance assistance ‘appropriately’, whereas others delayed calling for help.

*It’s like when you ask what tablets they’re on. “What you on them for?” “The doctor gave me them”.’…‘they’re not taught enough or they don’t know enough [about their condition] to tell.***[FG2/P5]**

*....people won’t ring…if people are suffering asthma long term they know how to manage their own condition in the main and quite often don’t ring until they really can’t manage any further.***[FG3/P1]**

#### *Unmet patient expectations*

Participants found that some patients had clear ideas on the treatment they felt should be given and they expressed frustration when patients refused to travel to hospital when they, the clinician, believed this was needed. Others found that patients wanted or needed treatments, such as antibiotics or steroids, which they could not provide. Patients’ ideas of what to expect from ambulance clinicians was also perceived to be influenced by the media:

*I think patients have an expectation, certainly watching things on telly […] there’s a certain expectation for us to give oxygen. They don’t necessarily have an appreciation of things like any BTS* [British Thoracic Society] *guidelines but they expect things to happen or treatment to be given not just the assessment phase but also a mask being put over their face with a medicine to help them get better.***[FG3/P2]**

Sometimes patients put social concerns before their health.

*Yeah you know if you’re at home and you’re not happy and you still think they need to go to* [hospital] *and they say no I’m not going, half the time it’s because they can’t get back from the hospital. It’s nothing to do with what you’ve done as a service or anything.***[FG1/P1]**

#### *Patient barriers of language and diversity*

Some patients did not understand how to use a peak flow meter or its correct use was impaired by communication difficulties, due to language barriers, sensory impairment or mental capacity issues.

Just to pick out the issue around language and communication, do you have access to language line or some sort of…?

An interpreter’s number.

And is that easy to access? Do you use it?

*No it’s not. I’ve used it. It’s a nightmare.***[FG1/Facilitator/P5/P1]**

*‘But the problem that you’ve got with peak flow is explaining it; unless you’re a regular user of the peak flow it’s very difficult to explain what you do.’ … ‘I think it’s a combination of things. It’s a combination of being hard of hearing. A lot of the elderly are hard of hearing. Having to explain it; people with learning difficulties, you’re going to struggle to explain.*’ **[FG3/P1]**

### Complexity of ambulance service care

#### *Difficulty of diagnosing or confirming asthma prehospitally*

There was a perception that some patients labelled by other healthcare professionals with asthma did not fit the diagnosis or that patients identified as asthmatic by the ambulance call and dispatch systems turned out on arrival not to be having an asthma attack. The presence of comorbidities also made assessment more difficult. It was suggested that algorithms used in call and despatch systems needed improvement to more clearly distinguish between possible asthma and other conditions.

*The first thing you need to establish is whether it is asthma. A lot of cases we go to are called asthma and when we get there you find that they’re actually probably a panic attack of some sort or something totally unrelated to asthma.***[FG1/P3]**

…having elderly people with standalone asthma, you don’t see it that often, do you.

*No, there’s often lots of comorbidity…’***[FG3/P3/P2]**

#### *Complex prehospital care processes*

The complexity of care and time pressures in the prehospital environment sometimes led to clinicians not recording observations, either because the patient and paperwork was handed to another crew before the opportunity to record them arose, or because assessments were made but clinicians forget to record them because of pressures of time or the situation.

*…items sometimes get missed… [data entry] boxes get missed off or not ticked or not written in because we’re doing so much....as we were talking about, being on the cars we’re doing so much and writing at the same time. Sometimes we will miss one box off and usually it’s that box we miss off because we haven’t had a chance to do it. Or we’ve done it and the crews come along and we want to get that patient to hospital.***[FG2/P1]**

#### *Equipment burden*

The weight and quantity of equipment needed to be taken into patients’ homes was also a problem, particularly for solo responders. Participants referred to the number of bags which needed to be carried, including equipment, drug bags, oxygen, and monitor/defibrillator which are stored separately, to ensure everything was to hand when treating the patient or in case of respiratory or cardiac arrest.

*You’re walking around the estate at two o’clock in the morning, looking for the patient and you’re literally carrying as much as you can. You physically cannot go back and get equipment.***[FG2/P4]**

### Improved education, information, communication, support and pathways

#### *Practitioner education and patient information*

Education and training of clinicians was discussed in general terms, rather than relating specifically to asthma, and was considered to be fundamental to improving care, but there was little consensus on the best methods for their delivery. It was felt that locally accessible clinical training was important and some, particularly longer serving paramedics who were trained in-house by ambulance services, felt they lacked the level of knowledge of clinicians who had qualified through university degree courses. Some participants acknowledged self-learning as a method of updating their skills and knowledge.

*It gets very difficult for the likes of us as PTLs to actually talk to a paramedic when they actually know things that you haven’t been taught.’***[FG1/P5]**

*I prefer actually being taught. I think that is one of the problems that we’re having, we are moving away from course orientated training to self-training.***[FG1/P8]**

#### *Interprofessional communication and clinical support*

Where clinicians had reached the limits of the treatment they are able to provide, support and advice from senior colleagues, or from other healthcare professionals, was also seen as important to improving care.

*There are times when we have reached our ceiling. We know that that patient needs A, B and C and we can’t do it. It’s not within our remit so our hands are tied and it’s incredibly frustrating. So…this is where a good* [Medical Director] *would come in and say ok well why don’t you give it if you’re really in that situation call me…there’s my telephone number in an emergency call us and I can authorise some more.***[FG2/P4]**

Better communication between ambulance staff, other healthcare professionals and patients was felt to be needed. Information provided to patients in the form of patient information leaflets on managing their asthma or self-care records which detailed normal PEFR levels and advice on self–management were considered to be of value. Patients and the practitioners caring for them could be reassured by ambulance clinicians contacting patients later to review their condition where they had not been transported to hospital.

*There are one or two patients now depending on where they live will have cards telling them what their oxygen saturation is if they have a chronic condition. Not many asthmatics will be able to give you a card which tells you what their peak flow reading will be when they’re well.***[FG3/P2]**

*I’ve said I’m going to give you a ring in about an hour’s time to see how you’re doing.’***[FG1/P6]**

#### *Better care pathways*

Practitioners welcomed provision of care pathways for asthma but these were variable. Local pathways which enabled access to advice from a respiratory nurse, direct admission to a respiratory ward where necessary, and referral pathways to general practitioners (GPs) or community services for asthma were considered helpful, providing alternatives to hospital admission, follow up and necessary adjustments to long-term treatment regimens.

*I think pathways are important. If there are no pathways then the patient will call 999 the next time it happens rather than the correct person so we’re going to get regular callers.***[FG1/P8]**

## Discussion

### Main findings

This qualitative focus group study aimed to identify factors which might prevent or enable adherence to asthma guidelines through exploration of the attitudes, perceptions and beliefs of ambulance clinicians. Recognition and understanding of these factors may lead to more effective interventions to improve ambulance service care for patients with asthma. We found five overarching themes: guidelines should be more relevant to ambulance service care; there were barriers to appropriate assessment; the approach needed to address conflict between clinicians’ and patients’ expectations; the complexity of ambulance care reduced adherence; there were opportunities for improved prehospital education, information, communication, support, and pathways.

### Improving adherence to prehospital asthma guidelines

Guidelines for asthma, like other clinical guidelines, may be difficult for health workers to follow because of problems of credibility, applicability and practicality [26]. We found that barriers and facilitators operated at different levels of organisation, provider and patient and that determinants of practice fell into a number of domains such as health professional factors, patient factors, professional interactions, incentives and resources that have been previously identified as being important [17].

To change prehospital care for asthma to practice which conforms to guidelines is likely to be equally problematic because of professional and organisational constraints [27], the complexity of the ambulance setting, together with patient, equipment and pathway factors, which can all affect adherence with guidelines for urgent care of asthma [4,13,28]. By exploring solutions to the barriers to appropriate assessment, guidelines that are more relevant to ambulance clinicians could be developed, taking into account the conflict between clinicians and patients’ expectations for treatment and the complexity of ambulance service processes.

### Increasing relevance of prehospital asthma guidelines

Participants felt asthma guidelines needed to be made more relevant to the ambulance service setting. By exploring solutions to the barriers to appropriate assessment, guidelines that were more relevant to ambulance clinicians could be developed, taking into account the conflict between patients’ demands for treatment and compliance with advice, and the complexity of ambulance service processes and pathways.

Better communication between ambulance services, primary care and secondary care is needed to provide more integrated, accessible and appropriate pathways of care for patients with asthma. Our findings suggest that improved practitioner education which emphasises better communication skills with patients is needed to address the gap between patient expectations and the delivery of prehospital care.

There has been limited previous research on the implementation or effectiveness of asthma assessment and treatment in the ambulance service arena. The guidelines are therefore reliant on evidence derived from the primary care or hospital setting, where patients are treated in a more controlled and predictable environment than the ambulance setting, with access to a working environment with additional staff, equipment, lighting and scene control which is not comparable.

### Increasing objective assessment

Objective assessment, using PEFR and pulse oximetry, is emphasised in current guidance as important for assessing asthma severity and deciding on optimal treatment or the need for hospitalisation [6,7], but we found that clinicians prioritised treatment over assessments, undervalued baseline assessments, and were further deterred from undertaking measurements due to pressures of time, patient or equipment barriers, and emergency department staff not asking for this information.

Some paramedics mistakenly believed that clinical judgement was as reliable as objective measurements which were likely to be undertaken [4,13,29] leading to severity being underestimated [13]. National benchmarking of ambulance care of asthma over the past five years in the UK has shown that assessments were less likely to be carried out and slower to improve than treatments [30].

### Communication and asthma care pathways

Paramedics in our study felt that care for patients with asthma might be improved and hospital admissions reduced with access to advice and support for them in clinical situations which went beyond their training and protocols. They also thought that communication with a patient’s GP or community respiratory nurse, after treating and leaving a patient at home, could improve long-term management and reduce repeat attendances and admissions, particularly if this involved a personalised action plan as part of self-management education [7]. They considered that direct transportation of critically ill patients with asthma to a respiratory ward would improve care. Participants suggested similar communication pathways could be set up between ambulance services and GPs as there was concern that GPs may not be aware how many asthma related attendances a patient may be experiencing if they are not transported to hospital. This information could allow GPs to recognise more easily where a patient’s asthma was not adequately controlled.

#### *Implications for future research, practice and policy*

More evidence for asthma care needs to be generated in the ambulance service environment. For example, research is required to determine whether call and dispatch systems can more reliably identify asthma severity; recent studies suggest that improvements in dispatch coding may more reliably identify urgency of breathing problems and a more appropriate ambulance response [31,32]. Research is also required to determine whether assessments of peak flow and oximetry are as useful, reliable and valid in the prehospital setting. Although the importance of objective assessment of asthma severity is made clear in ambulance guidelines, there is a need to establish the risks and benefits in delaying treatment until assessment is carried out in this setting. Guidelines and training need to emphasise the value and importance of objective assessment as well as treatment. Recording systems need to be improved to help ambulance clinicians record assessments and treatment or, at least, ensure that reasons for not carrying these out are recorded. Ways of improving the patient pathway and patient outcomes for asthma need to be explored further: the pathway for care may be developed by strengthening links between ambulance services, primary care, and secondary care or by implementing nationally standards for asthma pathways. This study looked at emergency prehospital asthma care purely from the perspective of ambulance clinicians and it may be useful to extend the study to study the understanding of ambulance service asthma care from the point of view of other healthcare professionals and patients.

#### *Strengths and limitations*

This study was conducted in a single UK regional ambulance service which may mean that some of the barriers and facilitators found are not generalisable to other services. However, all UK ambulance trusts follow JRCALC guidelines [6] and the national indicators for UK ambulance services show broadly similar deficiencies in care for asthma across services [14],which suggests that our findings may be applicable more widely. Since this study was completed JRCALC guidelines 2013 have been published [33]. Although these provide more detailed and up-to-date guidance, the evidence for them is still largely derived from research in acute and primary care settings. Both authors were based and involved in supporting clinical audit and research in the organisation which affected the formulation of the research question, encouraged discussion in the focus groups and supported the analysis.

## Conclusion

This qualitative study provides insight into paramedics’ perceptions of the assessment and management of asthma, including why paramedics may not always follow guidelines for assessment or management of asthma. These findings provide opportunities to strengthen clinical support, patient communication, information transfer between professionals and pathways for prehospital care of patients with asthma.

### Ethical approval

Ethical approval was obtained from the NHS Research Ethics Service (10/H0408/59). Approval for Research Management and Governance was sought and gained from NHS Lincolnshire and East Midlands Ambulance Services NHS Trust.

### RATS guidelines

The study adheres to RATS qualitative research guidelines.

## Competing interests

The authors declare that they have no competing interests.

## Authors’ contributions

DS and ANS conceived the original idea for the study. DS supported by ANS developed the study design. DS undertook the focus groups and analysis. DS wrote the first draft of the paper. All authors contributed to the discussion and final paper. DS is guarantor for the study. Both authors read and approved the final manuscript.

## Pre-publication history

The pre-publication history for this paper can be accessed here:

http://www.biomedcentral.com/1471-227X/14/18/prepub

## Supplementary Material

Additional file 1 Focus group topic guide. Click here for file

## References

[B1] Simpson CR Sheikh A Trends in the epidemiology of asthma in England: a national study of 333,294 patients J R Soc Med 2010 103 98 106 20200181 10.1258/jrsm.2009.090348 PMC3072257

[B2] Asthma UK Asthma facts and FAQs 2012 [http://www.asthma.org.uk/asthma-facts-and-statistics]

[B3] Smith SM Mitchell C Bowler SD Heneghan C Perera R The health behaviour and clinical characteristics of ambulance users with acute asthma Emerg Med J 2009 26 187 192 19234010 10.1136/emj.2008.059188

[B4] Davies BH Symonds P Mankragod RH Morris K A national audit of the secondary care of “acute” asthma in Wales–February 2006 Respir Med 2009 103 827 838 19200707 10.1016/j.rmed.2008.12.020

[B5] Simpson AJ Matusiewicz SP Brown PH McCall IA Innes JA Greening AP Compton GK Emergency pre-hospital management of patients admitted with acute asthma Thorax 2000 55 97 101 10639524 10.1136/thorax.55.2.97 PMC1745682

[B6] Joint Royal Colleges Ambulance Liaison Committee, Ambulance Service Association UK Ambulance Service Clinical Practice Guidelines 2006 London: Ambulance Service Association

[B7] British Thoracic Society, Scottish Intercollegiate Guidelines Network British guideline on the management of asthma Thorax 2008 63 4 iv1 iv121 18463203 10.1136/thx.2008.097741

[B8] Harrison B Stephenson P Mohan G Nasser S An ongoing confidential enquiry into asthma deaths in the eastern region of the UK, 2001–2003 Prim Care Respir J 2005 14 303 313 16701746 10.1016/j.pcrj.2005.08.004 PMC6743601

[B9] de Ribeiro AC Duarte MC Camargos P Correlations between pulse oximetry and peak expiratory flow in acute asthma Braz J Med Biol Res 2007 40 485 490 17401491 10.1590/s0100-879x2007000400006

[B10] Rodrigo G Rodrigo C Assessment of the patient with acute asthma in the emergency department: a factor analytic study Chest 1993 104 1325 1328 8222781 10.1378/chest.104.5.1325

[B11] Rodrigo G Rodrigo C A new index for early prediction of hospitalization in patients with acute asthma Am J Emerg Med 1997 15 8 13 9002561 10.1016/s0735-6757(97)90039-5

[B12] Rodrigo GJ Predicting response to therapy in acute asthma Curr Opin Pulm Med 2009 15 35 38 19077703 10.1097/MCP.0b013e32831da852

[B13] Snooks H Halter M Palmer Y Booth H Moore F Hearing half the message? a re-audit of the care of patients with acute asthma by emergency ambulance crews in London Qual Saf Health Care 2005 14 455 458 16326794 10.1136/qshc.2004.012336 PMC1744100

[B14] Shaw D Siriwardena AN Report on the National Ambulance Service Clinical Performance Indicators: Cycle 5 May 2010 - September 2010 2010 Nottingham: East Midlands Ambulance Service NHS Trust

[B15] Siriwardena AN Shaw D Donohoe R Black S Stephenson J Development and pilot of clinical performance indicators for English ambulance services Emerg Med J 2010 27 327 331 20385697 10.1136/emj.2009.072397

[B16] Cabana MD Rand CS Powe NR Wu AW Wilson MH Abboud PA Rubin HR Why don’t physicians follow clinical practice guidelines? a framework for improvement JAMA 1999 282 1458 1465 10535437 10.1001/jama.282.15.1458

[B17] Flottorp SA Oxman AD Krause J Musila NR Wensing M Godycki-Cwirko M Baker R Eccles MP A checklist for identifying determinants of practice: a systematic review and synthesis of frameworks and taxonomies of factors that prevent or enable improvements in healthcare professional practice Implement Sci 2013 8 35 23522377 10.1186/1748-5908-8-35 PMC3617095

[B18] Robertson R Jochelson K Interventions That Change Clinician Behaviour: Mapping the Literature 2010 London: NICE

[B19] Silverman D Qualitative Research: Theory, Method and Practice 2004 2 London: Sage Publications

[B20] Reeves S Albert M Kuper A Hodges BD Why use theories in qualitative research? BMJ 2008 337 a949 18687730 10.1136/bmj.a949

[B21] East Midlands Ambulance Service NHS Trust About us. EMAS website 2014 http://www.emas.nhs.uk/about-us/ [accessed 20.3.14]

[B22] Kreuger R Focus Groups: A Practical Guide for Applied Research 1994 London: Sage Publications

[B23] QSR International Pty Ltd NVivo Qualitative Data Analysis Software 2008 Version 8

[B24] Ritchie J Spencer L Bryman A, Burgess RG Qualitative Data Analysis for Applied Policy Research Analysing Qualitative Data 1994 London: Routledge 173 194

[B25] Pope C Ziebland S Mays N Qualitative research in health care: analysing qualitative data BMJ 2000 320 114 116 10625273 10.1136/bmj.320.7227.114 PMC1117368

[B26] Wiener-Ogilvie S Pinnock H Huby G Sheikh A Partridge MR Gillies J Do practices comply with key recommendations of the British asthma guideline? if not, why not? Prim Care Respir J 2007 16 369 377 18026674 10.3132/pcrj.2007.00074 PMC6634244

[B27] Snooks HA Kearsley N Dale J Halter M Redhead J Foster J Gaps between policy, protocols and practice: a qualitative study of the views and practice of emergency ambulance staff concerning the care of patients with non-urgent needs Qual Saf Health Care 2005 14 251 257 16076788 10.1136/qshc.2004.012195 PMC1744057

[B28] Pinnock H Johnson A Young P Martin N Are doctors still failing to assess and treat asthma attacks? an audit of the management of acute attacks in a health district Respir Med 1999 93 397 401 10464821 10.1053/rmed.1999.0575

[B29] van Wamel A Proctor S Why take a peak flow in asthma - a review J Paramed Prac 2010 2 56 62

[B30] Shaw D Siriwardena AN Report on the National Ambulance Service Clinical Performance Indicators: Cycle 7 June - September 2011 2011 Nottingham: East Midlands Ambulance Service NHS Trust

[B31] Clawson J Olola C Heward A Patterson B Scott G Profile of emergency medical dispatch calls for breathing problems within the medical priority dispatch system protocol Prehosp Disaster Med 2008 23 412 419 19189610 10.1017/s1049023x00006142

[B32] Clawson J Barron T Scott G Siriwardena AN Patterson B Olola C Medical priority dispatch system breathing problems protocol key question combinations are associated with patient acuity Prehosp Disaster Med 2012 27 375 380 22824188 10.1017/S1049023X1200101X

[B33] Joint Royal Colleges Ambulance Liaison Committee UK Ambulance Services Clinical Practice Guidelines 2013 2013 Bristol: Class Professional Publishing Ltd

