# Supplementary material for: Identifying barriers and facilitators to ambulance service assessment and treatment of acute asthma: a focus group study
Source: BMC Emerg Med. 2014 Aug 3;14:18. doi: 10.1186/1471-227X-14-18 (PMC4125344; doi:10.1186/1471-227X-14-18)
Supplement: Additional file 1 — Focus group topic guide. [file 1471-227X-14-18-S1.docx]

**Additional file 1: Focus Group topic guide**

**[Ambulance Trust Heading]**

**Researcher’s Focus Group Topic Guide:**

**Identifying barriers and facilitators to evidence based assessment of asthma: exploring the perception and belief of ambulance paramedics to the assessment of asthma**

**Experience and extent of the problem**

**Q.1. Can you please describe your experience when attending patients with**

**acute asthma?**

Prompts:

- what is your experience of assessing (undiagnosed or recurrent) asthma on

arrival at the scene?

- what do you feel/think patients expect from you on arrival at the scene?

(what sort of support/things do you think patients expect from you?)

- to what extent do patients expect you to manage the condition in the

ambulance/at home?

- to what extent are you able to manage asthma in the ambulance/at home?

**Assessment of asthma**

**Q.2. Can you describe your approach to assessing asthma?**

Prompt:

- how do you normally assess acute asthma in the home/ambulance?

- what factors do you take into consideration to assess the condition?

(history, equipment, vitals, any tests, SATS)

- what are the other things that actually help you to make an assessment?

(calming down, reassure, history from carer/family, patient’s medicine)

- what are the signs and symptoms or features that you look for to assess

the condition?

**Q.3. What device(s) do you use to assess asthma?**

Prompt:

- we are interested in SPO2 and Peak Flow

- what are the advantages of using these devices to facilitate your assessment?

- are there any difficulties that you may have come across while using these devices?

- what are your suggestion(s) to overcome the difficulties in making use of the devices?

devices?

**Q.4. Tell me your views on peak flow measurement?**

- what do you see as the benefits of doing a peak flow?

- what drawbacks are there of taking/not doing the peak flow?

- if peak flow is not being taken, can you tell me some of the common reasons why

that is happening?

- what are your suggestions to overcome those difficulties?

- what is your impression about the peak flow performance rate of your trust?

- what are your suggestions to improve the performance rate?

**Q.5. What equipment(s) do you feel is necessary and that you would use to carry**

**out an assessment for asthma in the ambulance/at home?**

- what sort of equipment do you have or use at present to assess the condition (asthma)?

- what additional equipment do you need to carry out the assessment properly?

- is there any other equipment that you would like to assist you to carry out assessments

better in the ambulance?

**Assessment of asthma (continued)**

**Q.6. What features do you look for to elicit any change in the patient’s**

**condition?**

- what are the things that you would look for to know whether a patient’s condition is

getting any better or worse/deteriorating?

- what are the indications of patient’s condition getting worse?

- what are the indications of patient’s condition getting better?

**Guideline and pathways**

**Q.9. Can you share your experience regarding the guidelines and**

**pathways that you follow?**

- which guideline and/or pathway do you follow?

- how does the guideline help you to treat the patient in the ambulance/at home?

- how closely do you feel the guidelines are followed?

- what difficulties did you come across in using the guideline (if any)?

- are there any suggestions or needs in overcoming these difficulties?

- what would be the optimum management for acute asthma in the prehospital setting

from your point of view as paramedic?

**Suggestions for improvement**

**Q.10. Can you summarise and list the common factors that you feel is important**

**in relation to improving care for asthma in [name of ambulance service]?**

- in your opinion, what are the things that need to improve for you to provide better

asthma care in the ambulance or at home?

(patient – awareness raising & motivation, paramedics – assessment & treatment,

organisation – equipment & training)

- what sort of back up support do you think could be of help to you?

- do you want to suggest any other thing needed relating to training, ways of giving

message and to update information on management of asthma in prehospital setting?

- are there any other factors you would like to suggest to improve the quality of asthma

care in ambulances within existing resources?

--------------------------------------------------------------------------
